# The AirSR two-component system contributes to *Staphylococcus aureus* survival in human blood and transcriptionally regulates *sspABC* operon

**DOI:** 10.3389/fmicb.2015.00682

**Published:** 2015-07-03

**Authors:** Jeffrey W. Hall, Junshu Yang, Haiyong Guo, Yinduo Ji

**Affiliations:** Department of Veterinary and Biomedical Sciences, College of Veterinary Medicine, University of Minnesota, St. Paul, MNUSA

**Keywords:** *S. aureus*, V8 protease, staphopain B, transcriptional regulation, AirSR (YhcSR)

## Abstract

To date, genes identified and transcriptionally regulated by the AirSR TCS have been involved in energy production and cellular homeostasis of the staphylococcal cell. It is well accepted that the state of cellular metabolism impacts the expression of virulence factors in *Staphylococcus aureus.* For this reason, we conducted experiments to determine if the AirSR TCS contributes to the pathogenesis of *S. aureus* using an antisense RNA interference technology, an inducible overexpression system, and gene deletions. Depletion of AirSR by antisense RNA expression or deletion of the genes, results in significant decrease in bacterial survival in human blood. Conversely, overexpression of AirR significantly promotes survival of *S. aureus* in blood. AirR promotes the secretion of virulence factors that inhibits opsonin-based phagocytosis. This enhanced survival is partially linked to the transcriptional regulation of the *sspABC* operon, encoding V8 protease (SspA), staphopain B (SspB) and staphostatin B (SspC). SspA and SspB are known virulence factors which proteolytically digest opsonins and inhibit killing of *S. aureus* by professional phagocytes. This is the first evidence linking the AirSR TCS to pathogenesis of *S. aureus*.

## Introduction

*Staphylococcus aureus* accounts for approximately 20% of bloodstream infections in the U.S. (Wisplinghoff et al., 2004). The bacteria gain access to the bloodstream commonly from the result of puncture wounds of the skin (Saravolatz et al., 1982; Control Centers for Disease Control and Prevention [CDC], 2003; Begier et al., 2004), surgical site infections, or insertion of central venous lines and catheters (Maki et al., 1997; Wisplinghoff et al., 2004). Once *S. aureus* enters the bloodstream, the bacteria have the ability to enter almost any site of the human body (Gordon and Lowy, 2008). *S. aureus* bloodstream infections often lead to septic shock and endocarditis (Lowy, 1998). Bacteremia was responsible for 75% of invasive *S. aureus* infections, which were identified by the Active Bacterial Core Surveillance program, a nationwide observation program of federal and state health officials. Septic shock and endocarditis accounted for an additional 10% of invasive infections (Klevens et al., 2007).

The pathogenicity of *S. aureus* partially relies on the coordinately regulated expression of virulence factors that allow the bacterium to evade the host immune system and/or promote survival during infection. Similar to other bacterial pathogens (Crosa, 1997; Cotter and DiRita, 2000; Ollinger et al., 2008; Tomaras et al., 2008; Hammerstrom et al., 2011; Ouyang et al., 2011), *S. aureus* has evolved a series of regulatory effectors (Crosa, 1997; Howell et al., 2003; Torres et al., 2007; Zheng et al., 2007; Montgomery et al., 2010) which allow the organism to sense and to adapt to changing environmental stimuli and survive within a particular niche by modulating specific cellular responses and virulence gene expression. Sixteen two-component systems are encoded in the core *S. aureus* genome, with many of them influencing the expression of virulence factors (Novick et al., 1993; Brunskill and Bayles, 1996; Giraudo et al., 1999; Fournier et al., 2001; Kuroda et al., 2003; Liang et al., 2005; Toledo-Arana et al., 2005; Meehl et al., 2007; Watkins et al., 2011, 2013). Some of these TCSs link cellular metabolism and virulence factor expression to the availability of extracellular nutrients, such as KdpDE and HssRS systems that sense extracellular K^+^ and heme, respectively (Torres et al., 2007; Xue et al., 2011). Analysis of AirSR to date has shown the two-component system to be a sensor of oxygen (Sun et al., 2011) that modulates the expression of pathways responsible for dissimilatory nitrate reduction (Yan et al., 2011), cellular osmotic balance (Yan et al., 2009) and alternative sugar catabolism pathways (Yan et al., 2012). Moreover, the AirSR TCS is important for aerobic and anaerobic growth of *S. aureus* (Sun et al., 2005; Hall and Ji, 2013).

The mechanisms by which *S. aureus* survives and subverts the vertebrate immune system have been studied for many decades. *S. aureus* produces various immune suppression factors, including V8 protease (*sspA*, serine endopeptidase), staphopain B (*sspB*, cysteine endopeptidase) and staphostatin B (*sspC*, inhibitor of Staphopain B). These proteases have been linked to a wide variety of innate immune system suppression pathways by their ability to degrade complement components (Jusko et al., 2013), induce vascular leakage and promote extracellular matrix structural damage (Imamura et al., 2005; Ohbayashi et al., 2011). In addition, the proteases inhibit neutrophil chemotaxis and induce apoptosis of neutrophils or engulfment of neutrophils by macrophages (Smagur et al., 2009a,b). The circulating neutrophils and monocytes are key innate cellular components to combat infection by *S. aureus* (Kapral and Shayegani, 1959; Mandell, 1974; Fournier and Philpott, 2005; DeLeo et al., 2009; Rigby and DeLeo, 2012; Spaan et al., 2013).

In this study, we found that the overproduction of AirR resulted in enhanced survival of *S. aureus* in human blood and inhibited opsonin-mediated phagocytosis. We identified that AirSR activates expression of the *sspABC* protease operon. Analysis of an *sspAB* mutant revealed the proteases are only one of many, as yet unidentified, proteins that contribute to AirSR-mediated survival in blood and inhibition of opsonophagocytic clearance of the bacteria.

## Materials and Methods

### Bacterial Strains, Plasmids, and Growth Media

The bacterial strains and plasmids used in this study are listed in **Tables 1** and **2**. The *S. aureus* cells were cultured in trypticase soy broth (TSB) at 37°C with shaking. *Escherichia coli* strains were grown in Luria-Bertani (LB) broth. Transformants containing recombinant plasmids were selected on LB agar containing ampicillin (100 μg/ml), kanamycin (50 μg/ml), or erythromycin (300 μg/ml) for *E. coli*, and trypticase soy agar (TSA) containing chloramphenicol (10 μg/ml), tetracycline (5 μg/ml), and/or erythromycin (5 μg/ml) for *S. aureus*. All overnight cultures grew to similar OD600_nm_ values.

**Table 1:** Bacterial strains.

Strain	Description	Reference
DC10B	Dam*^-^ Escherichia coli* which allows for direct electroporation of purified plasmid DNA into wild-type *Staphylococcus aureus*	Monk et al. (2012)
BL21(DE3)	*E. coli* used for protein expression; IPTG inducible; Cm^r^ and Kan^r^	Invitrogen
RN4220	Laboratory *S. aureus* strain (RsbU^-^)	Kreiswirth et al. (1983)
WCUH29	Encapsulated human clinical HA-MRSA isolate	NCIMB40771
AH1263	USA300 CA-MRSA Erm^s^ (LAC*)	(White et al., 2014)
AH2084	AH1263Δ*airSR*	White et al. (2014)
JE2	Plasmid cured derivative of LAC strain, community-acquired MRSA isolate	Fey et al. (2013)
MW2	USA300 Community acquired MRSA isolate	
MRSA923	Community-acquired MRSA isolate	
JSAS909	WCUH29 with pYJY909; Erm^r^	Sun et al. (2005)
WAirR	WCUH29 with pAirR; Erm^r^	This Study
JE2/pYH4	JE2 with pYH4; Erm^r^	This Study
JE2/pAirR	JE2 with pAirR; Erm^r^	This Study
MRSA923/pYH4	MRSA923 with pYH4; Erm^r^	This Study
MRSA923/pAirR	MRSA923 with pAirR; Erm^r^	This Study
MW2/pYH4	MW2 with pYH4; Erm^r^	This Study
MW2/pAirR	MW2 with pAirR; Erm^r^	This Study
NE1787	JE2 with *bursa aurealis* Tn in *srtA*	Fey et al. (2013)
NE1787/pYH4	NE1787 with pYH4; Erm^r^	
NE1787/pAirR	NE1787 with pAirR; Erm^r^	
WSspB	WCUH29 with pSspB; Erm^r^	This Study
Control P*ssp-lux*	WCUH29/pYH4 with P*ssp-lux*; Erm^r^	This Study
WAirR/P*ssp-lux*	WAirR with P*ssp-lux*; Erm^r^	This Study
AH1263/P*ssp-lux*	AH1263/with P*ssp-lux*; Erm^r^	This Study
AH2084/P*ssp-lux*	AH2084/with P*ssp-lux*; Erm^r^	This Study
ΔSspAB	WCUH29 with in-frame deletion of *sspAB*	This Study
ΔSspAB/pYH4	WCUH29Δ*sspAB* with pYH4; Erm^r^	This Study
ΔSspAB/pAirR	WCUH29Δ*sspAB* with pAirR; Erm^r^	This Study
ΔSspAB/pSspABC	WCUH29Δ*sspAB* with pSspABC; Erm^r^	This Study

**Table 2:** Plasmids.

Plasmids	Description	Reference
pCY1006	Shuttle vector carrying *agr* promoter-*gfp*-*lux* reporter, derives from pSB2019; Cm^r^, Amp^r^	Liang et al. (2006)
pET*airR*	pET24b based for production of AirR in *E. coli* BL21(DE3)	Yan et al. (2012)
pSAS909	pYH3 with *airSR* antisense downstream of TetR promoter; Amp^r^, Erm^r^	Sun et al. (2005)
pYH4	pYH3 with Amp^r^ removed; Erm^r^	Huang et al. (2004)
pAirR	*airR* cloned downstream of pYH4 TetR promoter for overproduction; Erm^r^	This Study
pSspB	*sspB* cloned downstream of pYH4 TetR promoter for overproduction; Erm^r^	This Study
P*ssp-lux*	*sspABC* promoter cloned upstream of promoterless *luxABCDE*; derived from pCY1006; Cm^r^, Amp^r^	This Study
pJB38-*sspAB*	pJB38 with in-frame *sspAB* upstream/downstream deletion region	White et al. (2014)
pSspABC	*sspABC* operon cloned downstream of pYH4 TetR promoter for overproduction; Erm^r^	This Study

### Blood Survival Assay

Strains were cultured in TSB with appropriate antibiotics. Inducer anhydrotetracycline (ATc) was added when indicated to overnight cultures. Following 18 h of culturing, the bacteria were washed twice in sterile PBS and suspended to an OD of 0.14 using a Behring photometer in PBS. Fresh venous human whole blood was collected using heparin containing Vacutainer tubes (BD) from outwardly healthy adult donors. The blood was then immediately used in the assay. Approximately 5 × 10^6^ CFU in 50 μl of PBS were added to 450 μl of blood per microcentrifuge tube with appropriate antibiotics and ATc, where indicated. Microcentrifuge tubes were capped and placed in a rotisserie incubator and incubated at 37°C with end-over-end mixing. At indicated time points a 20 μl sample was removed from each sample, serially diluted, and plated on TSA to determine the surviving CFU count for each sample. The percentage of surviving bacteria was calculated as CFU_timepoint_/CFU_initialinput_*100. Blood collection was approved by the University of Minnesota Institutional Review Board.

### Gene Deletion

Deletion of *sspAB* was carried out following the pKOR1 allelic exchange protocol as described (Bae and Schneewind, 2006) and primers in listed in **Table 3**. Plasmid pJB38 is a modified version of pKOR1 (Bose et al., 2013) and pJB38-*sspAB* was kindly provided by Alex Horswill (Mootz et al., 2013). All deletions were confirmed by diagnostic PCR.

**Table 3:** Oligonucleotide sequences^a^.

Primers	Sequence
ssp Pro For EcoRI	5′-TAGCGAATTCGATATGTTGAACT GGACGTCGTGAAC-3′
ssp Pro Rev XmaI	5′-TTCCCGGGCTAAAAACCTCCAA AAAATTTATTTACAAGTTAAATA TAACAC-3′
sspB-For	5′-AGGAGGTTTAAACTATGAATAGTTCATA TAAATCTAGAGTATTCA ATATTATAAGC-3′
sspB-Rev-AscI	5′-TT*GGCGCGCC*TTAGTAACC TATCATTGAACCATACCAG-3′
sspABC-For	5′-AGGAGGTTTAAACTATGAAAGGT AAATTTTTAAAAGTTAGTTC TTTATTCG-3′
sspABC-Rev-AscI	5′-TT GGCGCGCCTTATACTAAGCG CTCATAAACGATTGG-3′
AirROE-for	5′-AAACTATGGAACAAAGG ACGCGAC-3′
AirROE-rev	5′-TTGGCGCGCCCTATTTTA TAGGAATTGTGAATTG-3′
pJB38-ssp For	5′-GAATATGATATTAAGTC ACTTGCGTCG-3′
pJB38-ssp Rev	5′-GCTTATGAAATGGATGTT TTAAAAGAAGGTATG-3′
luxArev	5′-GCT CCA GTA ACC ATA CGG TAT C- 3′
TetRfor	5′-CAA TAC AAT GTA GGC TGC -3′
TTrev	5′-CTC AGG AGA GCG TTC AC -3′

*^a^Purchased from IDT DNA, Coralville, IA.*

### Cloning, Expression, and Purification of AirR-His Tagged Fusion Protein in *Escherichia coli*

The purification of AirR-6x His was carried out as described using the previously constructed pET*airR* plasmid (Yan et al., 2012). The only modification to the protocol was the use of Pro-Lyse^TM^ Bacterial Lysis Buffer (Lamda Biotech) to lyse the *E. coli*.

### SDS-PAGE Analysis of Exported Proteins, Mass Spectrometry Peptide-Protein Identification, and Immunoblotting

The culture supernatants were collected from the overnight cultures of *S. aureus* strains grown in TSB medium with 5 μg/ml of erythromycin and 250 ng/ml inducer ATc. Bacterial cells were pelleted by centrifugation at 3900 × g for 20 min. The culture supernatants were then passed through a 0.2 μm syringe filter to remove bacterial cells. The exported proteins were precipitated from an equal volume of supernatant using ethanol as described (Ji et al., 1999). The exported protein profiles were detected by 12% SDS-PAGE and Coomassie Blue staining. Prominent overproduced protein bands were cut from the gel and in-gel digested (Shevchenko et al., 1996). Samples were submitted to the University of Minnesota Mass Spectrometry Core for mass spectrometry. Immunoblotting for SspB was conducted as described previously (Liang et al., 2006) using a SspB antibody kindly provided by Alex Horswill (Mootz et al., 2013) and an alkaline phosphatase conjugated anti-chicken secondary antibody (Sigma). Overnight cultures grew to similar OD600_nm_ values and equal volume of precipitated protein from each culture supernatant was loaded rather than equal protein concentration so that differences in protein concentration could be observed.

### Zymography Analysis

Induced cultures were grown in TSB with appropriate antibiotics and 250 ng/ml of inducer ATc overnight at 37°C with shaking. The following day the bacterial cells were pelleted and the TSB culture supernatant was filter sterilized with a 0.2 μm syringe filter. Twenty five milliliters of each culture supernatant, along with sterile TSB as a vehicle control, were concentrated 50-fold using a Millipore Centrifugal Protein Concentrator with a 10 kD nominal molecular weight limit. Proteins were resolved using 12% SDS-PAGE, gelatin was added to a final concentration of 0.1% (v/v) for zymography analysis. An equal volume of each concentrated culture supernatant sample was mixed with protein solubilization buffer (5X, 50% glycerol, 10% (w/v) SDS, and 0.5 M Tris-HCl, pH 6.8) and incubated at room temperature for 30 min.

Each sample was loaded and resolved in the gelatin SDS-PAGE. After electrophoresis, the gel was placed in a plastic wash container and washed with 1X SDS removal buffer (2.5% Triton X-100, 5 mM MgCl_2_, 25 mM Tris-Cl, pH 7.5) for 60 min at room temperature. The SDS removal buffer was replaced after 30 min with fresh removal buffer and then rinsed gently with deionized (DI) water. Development buffer (0.1% Triton X-100, 5 mM MgCl_2_, 25 mM Tris-Cl, and pH 7.5) was added until it covered the gel and the container was incubated at 37°C overnight. After the development period, the development buffer was removed and the gel was rinsed gently with DI water. Stain buffer (50% DI water, 35% MetOH, 15% Glacial Acetic Acid, 0.25% Coomassie Blue R-250) was added to cover the gel in the container. The gel was incubated until it was no longer visible in the stain buffer. Stain buffer was removed and the gel was rinsed gently with DI water. Fixing buffer (2% Glacial Acetic Acid, 98% DI water) was added to the container until it covered the gel and incubated at room temperature for 24 h.

### Analysis of Transcriptional Regulation Using a Promoter-*luxABCDE* Reporter Fusion System

The upstream *ssp* promoter region was PCR amplified with primers ssp Pro For/ssp Pro Rev listed in **Table 3**, digested with *Eco*RI and *Xma*I (NEB) and replaced the *agr* promoter fragment in pCY1006. The re-constructed *ssp-lux* promoter reporter was confirmed by diagnostic PCR. Plasmids were purified from *E. coli* DC10B and electroporated into the *S. aureus* strains as indicated in **Table 1**. Bioluminescence intensity and optical density of the cultures were measured at different times of the experiment in duplicate. The Relative Light Units (RLU) were calculated by dividing the average bioluminescence reading by the average OD600_nm_ reading (lum/OD600_nm_) at each time point. The experiment was repeated three times with separate colonies of each strain.

### Construction of Overproduction Plasmids

Gene ORFs were obtained by PCR using Q5 high-fidelity polymerase (NEB) with the primers (AirROE-for/AirROE-rev; sspB-For/sspB-Rev-AscI; sspABC-For/sspABC-Rev-AscI) in **Table 3**. Purified PCR fragments were digested with *Asc*I. The pYH4 plasmid carrying the TetR regulated, ATc inducible promoter was digested with *Pme*I and *Asc*I. Digested PCR fragments were ligated into the digested pYH4 plasmid with T4 DNA ligase (Promega) and confirmed by diagnostic PCR using the Tetfor/TTrev primer pair listed in **Table 3** that are specific for regions upstream and downstream of the MCS in pYH4.

### HL-60 Opsonophagocytic Killing Assay

To determine the effect of *S. aureus* strains exported proteins on the activity of serum antibodies and complement, induced cultures were grown in TSB with appropriate antibiotics and 250 ng/ml of inducer ATc overnight at 37°C with shaking. The following day the bacterial cells were pelleted and the TSB culture supernatant was filter sterilized with a 0.2 μm syringe filter. Twenty five milliliters of each culture supernatant, along with sterile TSB as a vehicle control, were concentrated 50-fold using a Millipore Centrifugal Protein Concentrator with a 10 kD nominal molecular weight limit. Before the HL-60 phagocytic assay, each concentrated culture supernatant and TSB was mixed 1:1 with the human serum or rabbit complement, incubated at 37°C for 30 min, and then placed on ice. In the assay, 40 and 20 μl of the serum mixture and complement mixture, respectively, were added to each well. Additional buffer was added to the complement mixture wells so all wells were of equal 100 μl volume.

HL-60 pluripotent cells were differentiated to granulocytic cells and cultured for 5 days as described (Kim et al., 2003). The basic assay consisted of 1,000 CFUs of *S. aureus* WCUH29 placed in duplicate of a 96 well microtiter plate. Pre-treated human serum and complement from 3 to 4 week of white rabbits (Life Technologies), respectively, were added to each well. Lastly, 4 × 10^5^ differentiated HL-60 granulocytes were added to each well to initiate the assay. The plates were incubated for 60 min at 37°C and with a CO_2_ concentration of 5%. Each well was mixed gently and 10 μl of sample from each well was drop plated in triplicate on TSA plates to determine surviving CFU. The percent survival was calculated as the number of surviving CFU/number of input CFU multiplied by 100, (CFU_f_/CFU_i_*100). The experiment was repeated at least 4 times.

### Statistical Analysis

Statistical data analysis was performed in Microsoft Excel for Mac 2011 using unpaired Student’s *t-*tests with a alpha level ≤ 0.05. Significant differences are noted by the addition of the *p-*value over the data being compared.

## Results

### AirSR Contributes to the Survival of *S. aureus* in Human Blood

**FIGURE 1:**
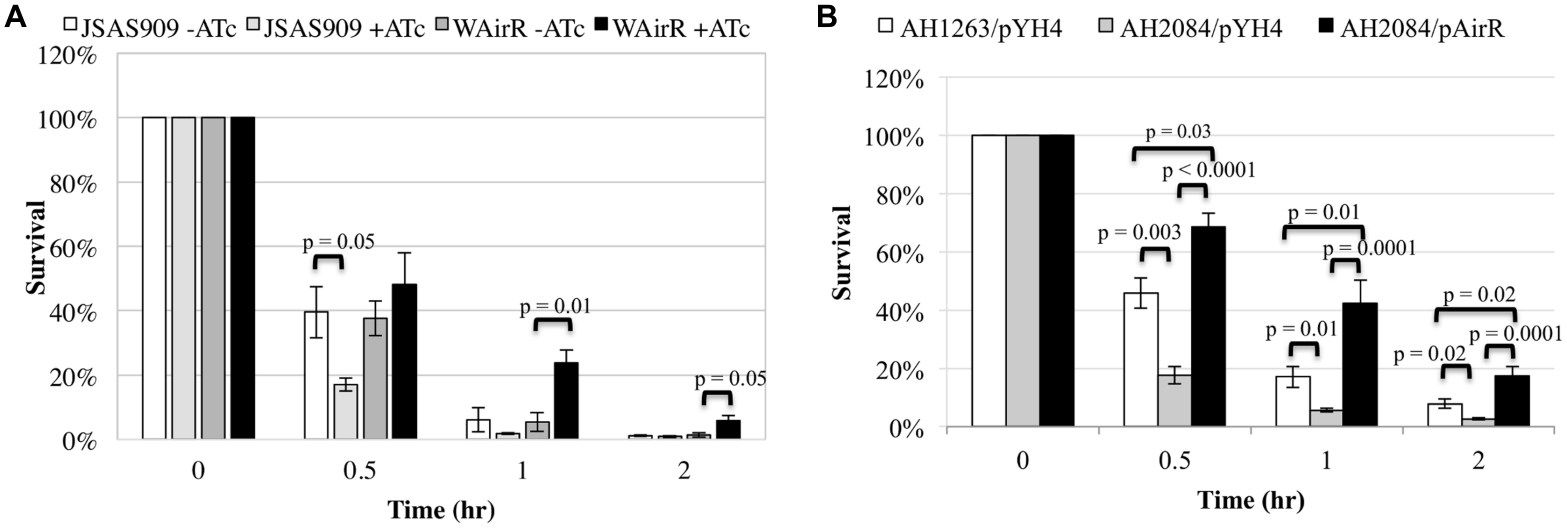
**The AirSR TCS is important for survival in human blood. (A)** Percent survival of the induced *Staphylococcus aureus airSR* antisense strain (JSAS909, 500 ng/ml ATc) and AirR overproduction strain (WAirR, 250 ng/ml ATc) in human blood during induction. **(B)** Percent survival of the wildtype LAC* (AH1263/pYH4), LAC*Δ*airSR* (AH2084/pYH4), and LAC*Δ*airSR* /AirR overproduction strain (AH2084/pAirR) in human blood during with 250 ng/ml of inducer ATc. Data represents the mean and SEM of at least three experiments.

The AirSR TCS is essential for growth in *S. aureus* WCUH29 (Sun et al., 2005), and it is important to validate the *in vivo* essentiality of any gene as some genes found to be essential *in vitro* may not be essential *in vivo* (Gandotra et al., 2007; Brinster et al., 2009). Survival of the *airS* antisense RNA strain (JSAS909) in human blood was examined as an initial step to determine the importance of *airSR* for survival the human host. An equal number of colony forming units (CFUs) per strain were inoculated into a defined volume of freshly isolated venous blood and depletion of AirSR by induction of *airSR* antisense RNA with ATc (Sun et al., 2005) resulted in a significantly decreased percentage of ATc induced JSAS909 CFUs surviving in the first half hour of incubation in human blood compared to the non-induced inoculum (**Figure 1A**, 18% vs. 40%). After 1 h, fewer ATc induced JSAS909 CFUs survived compared to the non-induced JSAS909, but was not statistically different. After 2 h of incubation, a similar percentage of CFUs survived for both strains (**Figure 1A**). Uninduced (–ATc) and induced (+ATc) empty plasmid control strains survived equally well (data not shown).

The induction of *airSR* antisense RNA, results in a delayed growth phenotype in WCUH29 (Sun et al., 2005). To eliminate impact of growth factors on bacterial survival in human blood, we determined if the overproduction of the AirR response regulator could promote survival in blood using an inducible overproduction strain (WAirR). ATc induced overproduction of AirR promoted survival of *S. aureus* WCUH29 over the course of the 3-h experiment compared to non-induced WAirR (**Figure 1A**). By hour two of the experiment, a significantly greater percentage of the initial inoculum of the induced WAirR strain survived in the blood compared to the uninduced WAirR strain (**Figure 1A**, 24% vs. 5%) and the increased survival of induced WAirR continued into hour three of the experiment.

To determine if the enhanced survival of *S. aureus* during AirR overproduction was applicable to other genetic backgrounds of *S. aureus*, we examined the effect of AirR overproduction in community-acquired methicillin resistant *S. aureus* (CA-MRSA) strains, MW2, MRSA923, and JE2. Similar to the results found with WCUH29, ATc induced overproduction of AirR greatly increased the percentage of CFUs that survived in human blood for all strains (Supplementary Figure S1).

Since the first publication on the identification and essentiality of *airSR* in strain WCUH29 (Sun et al., 2005), there have been several other research articles published investigating various aspects of the biological function of AirSR and the essentiality of *airSR* in other *S. aureus* strains has been disputed in these articles (Sun et al., 2011; White et al., 2014). This difference in essentiality may be due to distinct genetic differences between the strains of *S. aureus* used in each study. Most recently, the clean deletion of *airSR* was reported in *S. aureus* AH1263, a derivative of LAC (White et al., 2014). Following this publication, the pJB38-*airSR* deletion plasmid was introduced into both WCUH29 and *S. aureus* JE2, respectively. Approximately 100 colonies of WCUH29 and JE2 were screened for deletion of *airSR* using diagnostic colony PCR. Deletion of *airSR* was not detected in strain WCUH29 but was readily detected in the JE2 strain (data not shown), indicating the essentiality of *airSR* is strain dependent.

To determine if the *airSR* null deletion in AH1263 impacted bacterial survival in human blood, we conducted blood survival assays. Similar to our results with strain JSAS909, deletion of *airSR* significantly impaired the ability of AH1263/pYH4 to survive in whole blood (**Figure 1B**). The decreased survival of the AH2084/pYH4 strain was more than complemented by introduction and ATc induction of the AirR overproduction plasmid, with AH2084/pAirR having significantly enhanced survival relative to the AH1263/pYH4 and AH2084/pYH4 (**Figure 1B**). All three strains were assayed as group, thus the empty pYH4 control strain was introduced into the AH1263 and AH2084 to control for potential effects caused by the use of erythromycin and inducer ATc during the blood survival assay.

### AirR-Mediated Secreted Factors are Important for Enhanced Survival and Inhibited Opsonophagocytic Killing of *S. aureus* WCUH29

*Staphylococcus aureus* produces numerous LPXTG cell-surface linked MSCRAMMs and exported virulence factors involved in inhibition of complement and antibody mediated phagocytosis that enhance survival in blood and tissues (Zecconi and Scali, 2013; Foster et al., 2014). Since the JE2 strain showed similar enhanced survival to WCUH29, we utilized the *srtA* JE2 *bursa aurelis* Tn mutant, NE1787, to determine which surface factor(s) are involved in the enhanced survival in blood mediated by AirR. Sortase A is a transpeptidase responsible for proper LPXTG-MSCRAMM attachment to the cell surface. The AirR overproduction plasmid (pAirR) was electroporated into NE1787. We found Tn mutagenesis of *srtA* had no influence on AirR enhanced bacterial survival in human blood (data not shown), indicating SrtA processed MSCRAMMs are not responsible for the AirR-mediated enhanced survival in blood.

**FIGURE 2:**
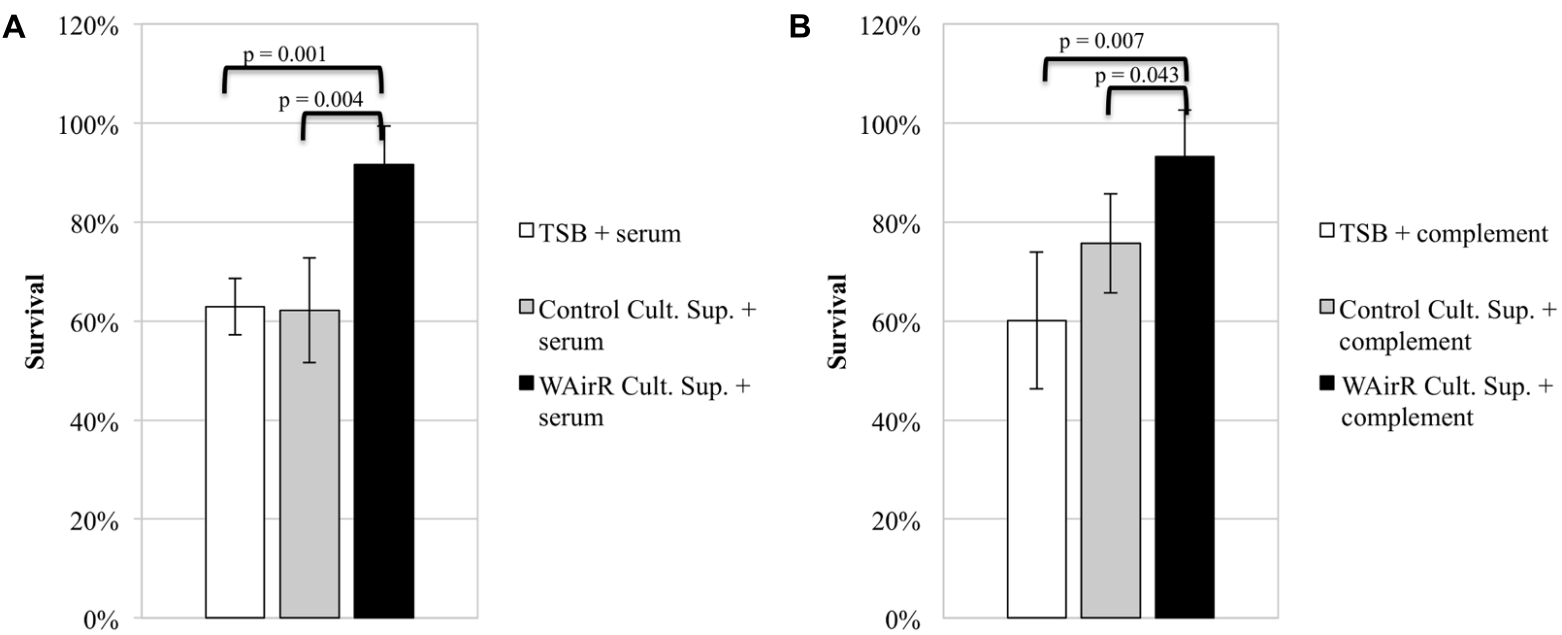
**AirSR regulated exported proteins inhibit complement and antibody mediated opsonophagocytic killing of *S. aureus*.** Sterile TSB and culture supernatants of ATc (250 ng/ml) induced control (Control Cult. Sup) and AirR overproducing *S. aureus* (WAirR Cult. Sup) were concentrated 50-fold. The serum **(A)** and complement **(B)** components of the assay were pre-incubated 30 min with concentrated sterile TSB or individual culture supernatants before addition to the assay. Data represents the mean and standard error of four individual experiments.

To investigate if exported proteins contribute to AirSR regulated anti-phagocytic mechanisms, we determined the effect of culture supernatants on bacterial anti-phagocytic capacity using a HL-60 opsonophagocytic killing assay (see Materials and Methods). If the induced WAirR gives rise to more anti-phagocytic virulence factors, a greater percentage of wild-type *S. aureus* WCUH29 CFUs would be expected to survive when the fractions are incubated with ATc induced WAirR culture supernatant compared to sterile concentrated TSB or concentrated ATc induced empty plasmid control supernatants. Indeed, significantly more wild-type *S. aureus* WCUH29 survived when the serum fraction (**Figure 2A**, 90% vs. 60%) or complement fraction of the assay (**Figure 2B**, 90% vs. 75%) was pre-incubated with concentrated induced WAirR culture supernatant compared to the induced control supernatant. As a control, concentrated TSB growth medium was included and did not impact the killing *S. aureus* relative to the induced control plasmid supernatant. These data suggest the AirSR two-component system contributes to *S. aureus* survival in human blood by promoting production of anti-opsonophagocytic virulence factors that inhibit serum- and complement-mediated mechanisms.

### Identification of Overproduced Exported Proteins Resulting from AirR Overproduction in *S. aureus*

To identify which exported protein(s) are overexpressed resulting from AirR overproduction, we prepared the exported proteins from cell-free culture supernatants of ATc induced pYH4 control and WAirR strains. The exported proteins were visually compared using SDS-PAGE, and protein bands that were obviously over-represented or bands that only appeared in the WAirR lane were processed for peptide identification by mass spectrometry (see Materials and Methods).

The subsequently identified proteins were cross-referenced with published studies to identify proteins that are involved in innate immune suppression via inhibition of the humoral and/or innate cellular response. Peptides from the cysteine endopeptidase, staphopain B (SspB) dominated one of the over-represented bands from ATc induced WAirR culture supernatants. More than 80% of the processed active form of SspB was identified by mass spectrometry (Supplementary Figure S2).

### AirR Overproduction Results in Increased Functional Staphopain B Production

**FIGURE 3:**
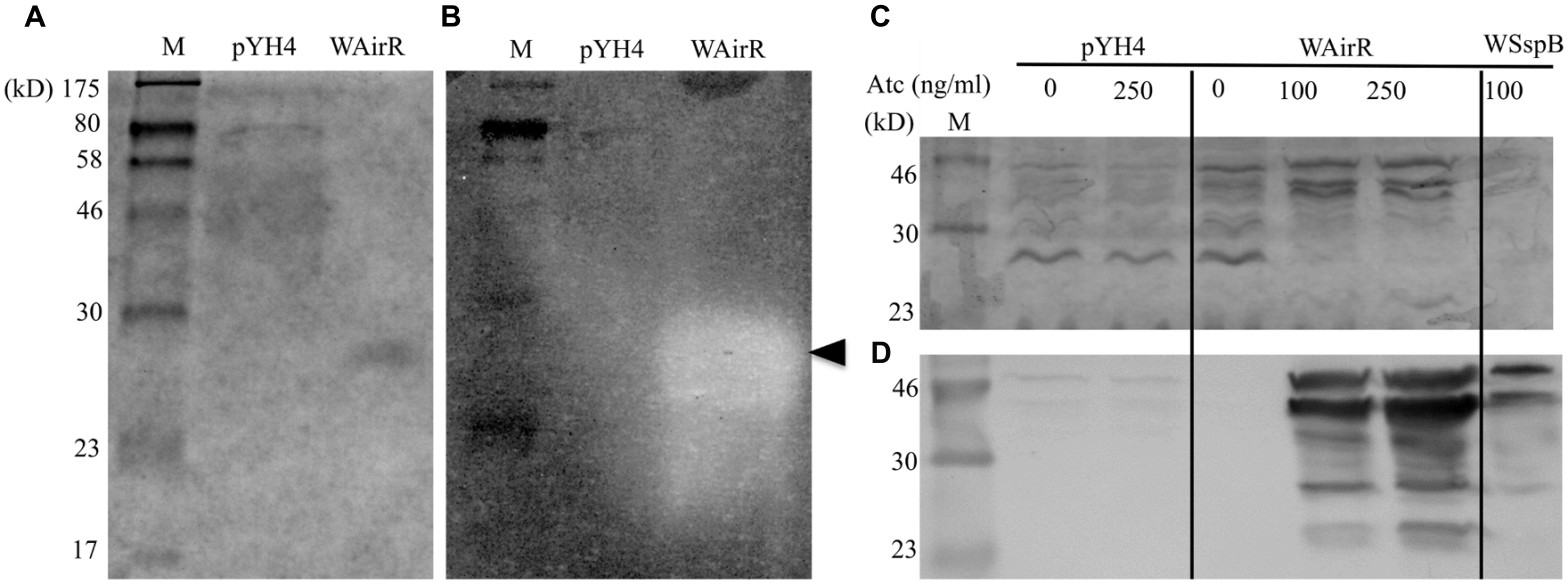
**Zymographic and immunoblot analysis of concentrated control and WAirR exported proteins**. Equal volumes of concentrated culture supernatant were resolved by **(A)** 12% SDS-PAGE and proteins stained with Coomassie Blue or **(B)** 0.1% gelatin-12% SDS-PAGE and processed for zymography, (←, indicates point of gelatin degradation). **(C)** SDS-PAGE analysis of precipitated culture supernatant proteins from control and WAirR strains without and with ATc and WSspB with ATc. **(D)** Immunoblot detection of SspB from precipitated culture supernatants showing increased amounts of SspB with increasing induction of AirR by inducer ATc.

To examine if the overproduced SspB is functional, we conducted gelatin zymography assays using the cell-free culture supernatants, as SspB is able to degrade collagen (Ohbayashi et al., 2011). Coomassie Blue staining and gelatin zymography analyzed was used to analyze an equal volume of concentrated culture supernatant from each induced strain. A single band in the induced WAirR lane and the disappearance of other proteins relative to the pYH4 control lane was detected by Coomassie Blue (**Figure 3A**). Gelatin zymography analysis of the same samples revealed very little gelatin degradation in the control strain, while a large, prominent band of gelatin degradation appeared in the induced WAirR sample (**Figure 3B**). Importantly, both the Coomassie Blue stained protein band and gelatin degradation in the zymogram resolve at the same molecular weight from the induced WAirR, suggesting the gelatin degradation is the result of this protein. These data highly suggest that the overproduction of AirR results in the overproduction of functional cysteine endopeptidase SspB.

To test the hypothesis that the increased gelatin degradation is the result of increased production of SspB and to confirm our mass spectrometry identification data, we ethanol precipitated the exported proteins from the cell-free culture supernatants of the pYH4 control and WAirR strains without and with inducer ATc. As seen in **Figure 3C**, the addition of ATc had no apparent impact on the protein profile of the control strain, while the addition of ATc to WAirR resulted in a stronger detection of a protein band similar in size to the SspB zymogen at 44 kD. Additionally, many protein bands were absent in the stained SDS-PAGE from the induced WAirR supernatant, consistent with previous reports that up-regulation of SspB (and SspA) results in degradation of other exported proteins (Karlsson et al., 2001; Jones et al., 2008). As a positive control, the *sspB* gene was cloned into the same ATc inducible expression vector (pSspB, strain WSspB). Further confirmation of SspB up-regulation was carried out by immunoblotting using chicken egg antibody specific for SspB (Kolar et al., 2013). In the control strain, SspB production was low and appeared unaffected by the addition of ATc (**Figure 3D**). In an ATc dose-dependent manner, staphopain B production was up-regulated in the WAirR supernatant (**Figure 3D**). SspB was readily detectable in the induced positive control WSspB supernatant as well. The SspB specific antibody detected the various processed and degraded forms of the protein (Shaw et al., 2005, 2004). These data clearly indicate a regulatory link between AirSR and SspB production.

### Transcription from the *ssp* Promoter is Regulated by AirR

**FIGURE 4:**
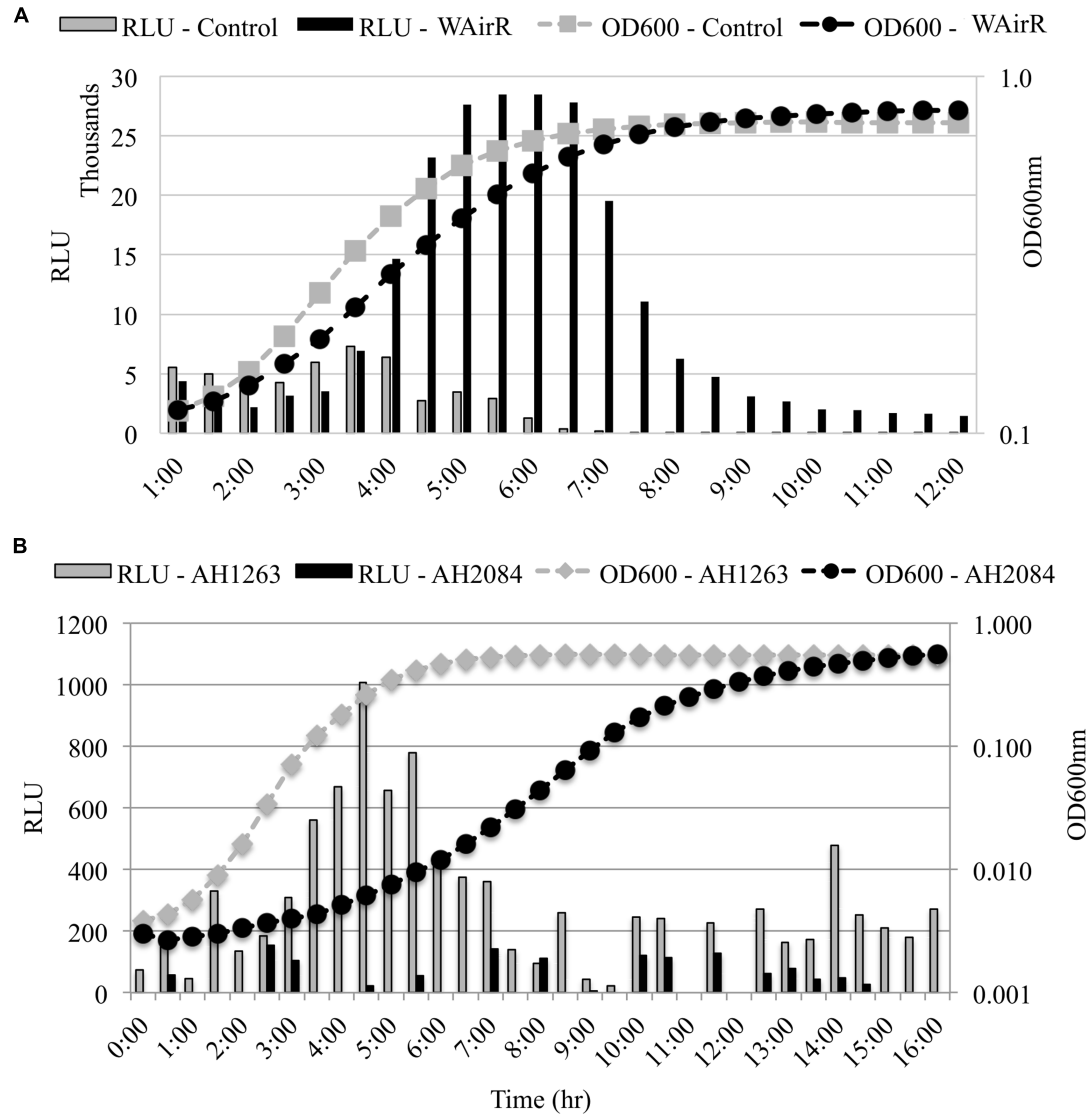
***ssp* promoter–reporter analysis**. All strains harbor the P*ssp-lux* reporter plasmid. **(A)** Uninduced overnight cultures of control and WAirR were diluted 1:300, incubated at 37°C with 25 ng/ml of inducer ATc. **(B)** Uninduced overnight cultures AH1263 and AH2084 were diluted 1:1000 and incubated at 37°C. OD600_nm_ and bioluminescence readings were measured every 30 min with 1 min of mixing before each reading in a BioTek Synergy II spectrophotometer. The light intensity values for each time point are given as relative light units (RLU), Lum reading/OD600_nm_ reading for each time point. Data presented are the mean of three independent colonies.

Staphopain B is produced from the middle gene of a three gene operon and is bordered upstream by *sspA*, encoding the V8 serine endopeptidase and downstream by *sspC* which encodes staphostatin B, a cytoplasmic inhibitor of SspB (Rice et al., 2001). To determine if the up-regulation of SspB production occurs post-transcriptionally or if transcription from the *ssp* promoter is increased in the induced WAirR strain, we examined the effect of AirR overproduction and deletion of *airSR* on the transcription of the *ssp* operon using a *ssp* promoter-*luxABCDE* reporter system. The induction of AirR production with inducer ATc resulted in a fivefold maximal increase in bioluminescence intensity compared to the control (**Figure 4A**). Furthermore, bioluminescence driven by the *ssp* promoter was higher and sustained throughout the growth of WAirR, demonstrating that continued and prolonged AirR overproduction results in increased transcription from the *ssp* promoter (**Figure 4A**). To examine if the absence of AirSR impacts the *ssp* promoter driven bioluminescence, the *ssp-lux* reporter was electroporated into the wild-type AH1263 and Δ*airSR*, AH2084, strains. Maximum *ssp-*driven bioluminescence was reduced fivefold in AH2084 compared to AH1263 (**Figure 4B**). These data indicate AirSR is a positive transcriptional regulator of the *sspABC* operon.

### SspABC is Not the Only Virulence Factor Involved in AirSR Mediated Survival in Human Blood

**FIGURE 5:**
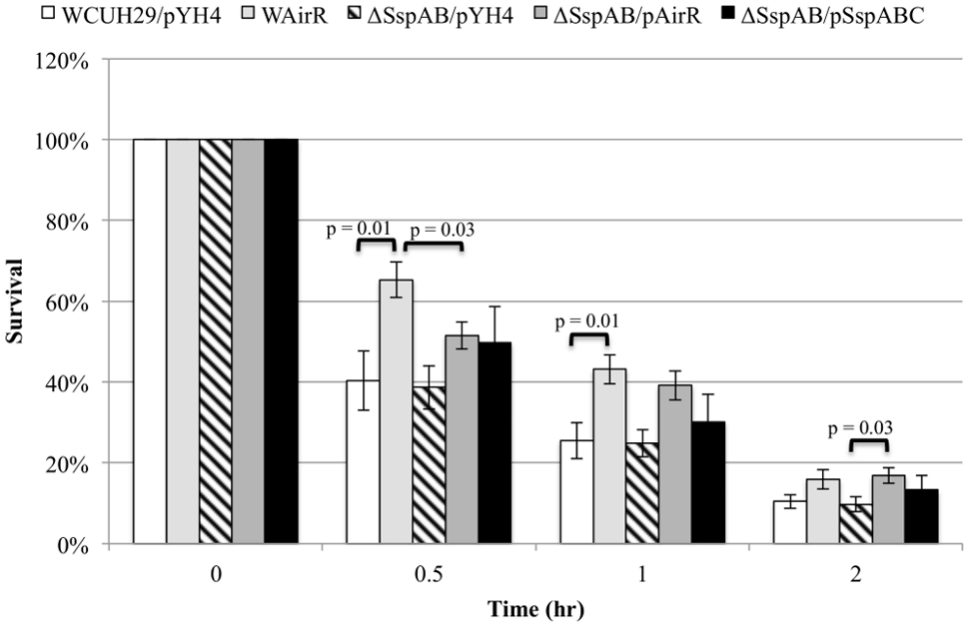
**Blood survival analysis of the wildtype and ΔSspABC without and with AirR and SspABC overproduction.** Percent survival of the wild-type *S. aureus* WCUH29 and ΔSspAB mutant strain with pYH4 control, pAirR, and pSspABC overproduction plasmids in human blood with 250 ng/ml inducer ATc. Data is the mean and SEM of at least three experiments per strain.

To investigate if AirR mediated enhanced survival and antiphagocytosis is due only to up-regulation of the Ssp proteases, we created a *sspAB* null mutant in *S. aureus* WCUH29 using an in-frame *sspAB* deletion plasmid (Mootz et al., 2013; kindly provided by Alex Horswill). We examined the survival of ATc induced wild-type WCUH29 and ΔSspAB without and with AirR overproduction in human blood. As seen previously, the overproduction of AirR increased the percentage of CFU that survived throughout the experiment (**Figure 5**, WCUH29/pYH4 vs. WAirR). However, deletion of *sspAB* did not result in decreased survival compared to wild-type WCUH29. The ΔSspAB/pAirR strain survived better compared to ΔSspAB/pYH4, but the percentage of surviving CFUs was statistically reduced when compared to induce WAirR in the first half hour of the assay only (**Figure 5**, WAirR vs. ΔSspAB/pAirR). After the first half hour, the deletion of *sspAB* had a minimal impact on the enhanced survival mediated by AirR overproduction and by two hours, WAirR and ΔSspAB/pAirR had similar percentages of surviving CFUs. Complementation of the ΔSspAB with an *sspABC* expression plasmid, on average, increased the percentage of bacteria that survived in human blood, but was not statistically different from WCUH29 or ΔSspAB. These data suggest, overall, SspAB contributes minimally to AirSR-mediated survival of *S. aureus* in human blood in the absence of AirR overproduction. Nonetheless, observing that the ATc induced ΔSspAB/pAirR strain had enhanced survival compared to ΔSspAB/pYH4 indicates additional, as yet unidentified virulence factors, are regulated by AirSR.

## Discussion

In this study, our data is the first to show that the AirSR two-component regulator is involved in pathogenesis of *S. aureus*. We utilized the inducible *airSR* antisense RNA and AirR overproduction approaches to alter the intracellular level of AirR and analyzed the impact of AirSR on bacterial survival and resistance to phagocytosis in healthy human whole blood. We revealed that the depletion of AirSR significantly inhibited the ability of the HA-MRSA isolate WCUH29 to survive in human blood during the first half hour of the assay in diverse staphylococcal genetic backgrounds, whereas the overproduction of AirR significantly enhanced survival in blood over 2 h. It was believed that *airSR* was essential for *S. aureus* growth, but this appears to a property of the WCUH29 strain. Recently, a Δ*airSR* mutant in the USA300 CA-MRSA lineage of *S. aureus* was constructed (White et al., 2014). Whereas *airSR* is not essential in this strain, the strain does appear to have a growth defect, as it grew much slower in the P*ssp-*reporter assay (**Figure 3B**), suggesting that AirSR, while dispensable in USA300 AH1263, is likely an important two-component system for *S. aureus* growth. These data indicate that the essentiality of *airSR* appears to be strain dependent. Similar to the WCUH29 *yhcSR* antisense RNA strain, the USA300 AH2084Δ*airSR* mutant survived significantly worse than the wild-type control (AH1263) in human blood. Furthermore, the overproduction of AirR in this mutant resulted in significantly enhanced survival of the strain in blood. These data clearly indicate, regardless of genetic background, the AirSR two-component system is important for survival in human whole blood. Additionally, analysis of a *srtA* mutant indicates that AirSR contributes to *S. aureus* survival in blood by regulating secreted factors, independent of cell wall-attached LPXTG-MSCRAMMS.

Our study indicates that one of these contributing AirR-regulated secreted factors is the cysteine endopeptidase, SspB, and possibly the serine protease, SspA, due to co-transcription. Our results show that AirSR mediates the production of staphopain B (SspB) using mass spectrometry, which was further supported by immunoblotting and gelatin zymographic assays. Moreover, using a promoter–reporter system it was demonstrated that AirR regulates the transcription of the *sspABC* operon, encoding V8 protease and staphopain B, both of which are known to promote survival in serum and inhibit opsonophagocytosis (Ryan et al., 2008; Smagur et al., 2009a,b; Jusko et al., 2013). This information corresponds well with the finding that the WAirR culture supernatant inhibits opsonophagocytic killing of *S. aureus* relative to the control extraordinarily well.

The AirSR TCS system regulates gene expression in response to the presence or absence of oxygen, and possibly reactive oxygen species (Sun et al., 2011). The regulation of *sspABC* by AirSR is of interest in the context of biofilm formation and stability and abscess formation, in addition to its apparent role in survival in blood. Biofilms and wound sites are known to have varying degrees of hypoxia (Sawyer et al., 1991; Beenken et al., 2004; Resch et al., 2005), thus, it is conceivable that AirSR may regulate expression of *sspABC* in response to the oxygen levels in the surrounding microenvironment. This regulation has implications in biofilm formation and stability, extracellular matrix destruction and wound healing, as well as neutrophil infiltration and immune response to infections (Imamura et al., 2005; Vincents et al., 2007; Smagur et al., 2009b; Ohbayashi et al., 2011; Chen et al., 2013; Jusko et al., 2013; Kolar et al., 2013; Mootz et al., 2013). Further investigation is needed to define the role of AirSR during systemic and abscess infections in relation to oxygen levels in these microenvironments and how AirSR regulation of *sspABC* and additional secreted virulence factors impacts the pathogenesis of *S. aureus* in these environments.

To elucidate whether the enhanced bacterial survival in human blood by overexpression of AirR is attributable to its positive regulation of the *sspABC* operon, we determined the impact of the *sspABC* null mutation on AirR-mediated anti-phagocytosis. We found the deletion of the *sspABC* operon did not significantly alter the survival capacity of wild-type WCUH29 strain, but did significantly reduce survival for the WAirR strain in the first half of the assay. Our studies indicate, as yet unidentified secreted AirSR regulated virulence factors, contribute to the ability *S. aureus* to resist phagocytosis and survive in human blood.

## Conclusion

The AirSR two-component system is involved in the modulation of *S. aureus* survival in human blood. The AirSR system positively regulates the expression of the *sspABC* operon at the transcriptional level and additional secreted virulence factors. Studies are ongoing to identify the additional factors that are regulated by AirSR, how oxygen impacts AirSR-mediated pathogenesis, and the contribution of these factors to anti-phagocytosis and pathogenesis of *S. aureus*.

## Conflict of Interest Statement

The authors declare that the research was conducted in the absence of any commercial or financial relationships that could be construed as a potential conflict of interest.
