# Supplementary material for: The AirSR two-component system contributes to Staphylococcus aureus survival in human blood and transcriptionally regulates sspABC operon
Source: Front Microbiol. 2015 Jul 3;6:682. doi: 10.3389/fmicb.2015.00682 (PMC4490255; doi:10.3389/fmicb.2015.00682)
Supplement: Supplementary file 1 [file Presentation_1.PDF]

Figure S1

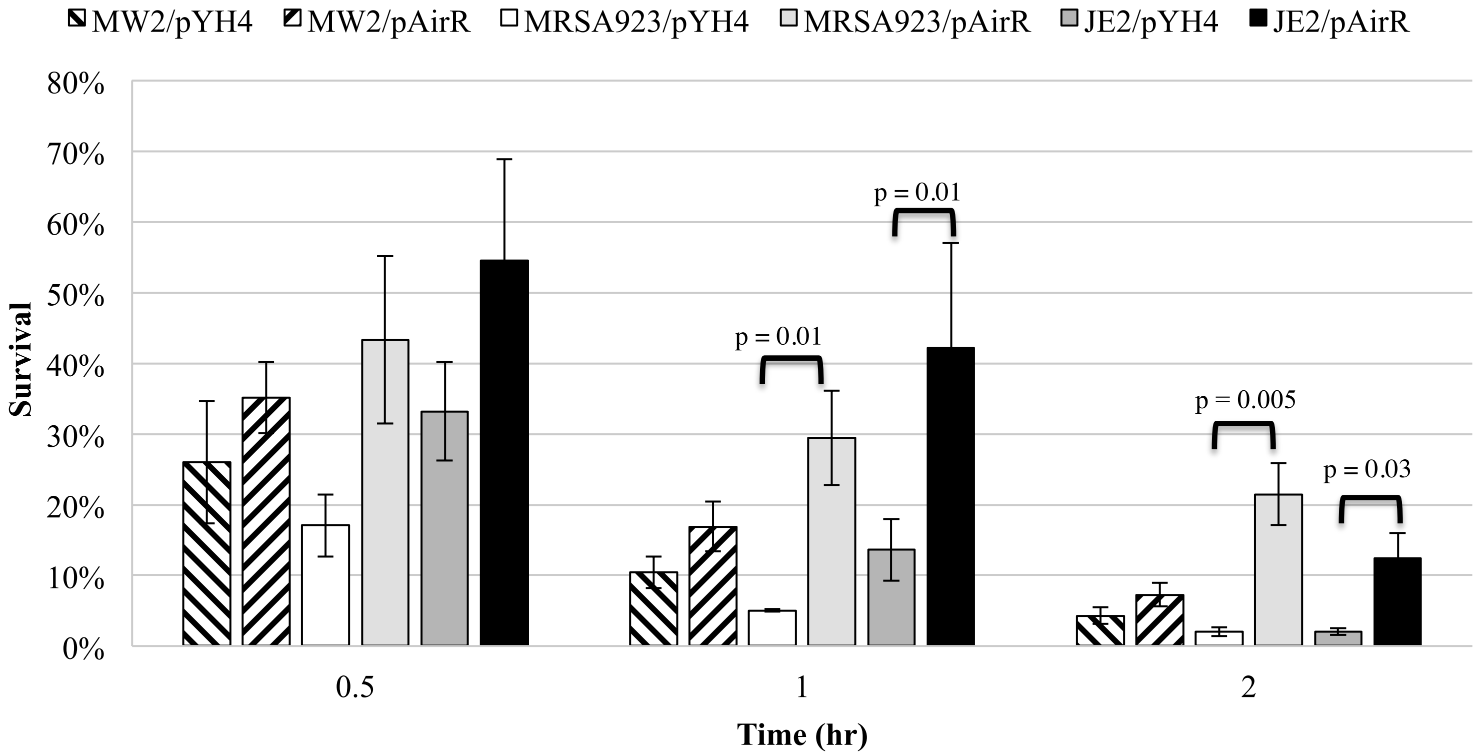

Figure S1. Overproduction of AirR contributed to survival of CA-MRSA in human blood. Percent survival of the CA-MRSA *S. aureus* (A) JE2 and (B) 923 during AirR overproduction. Data represents the mean and standard error of at least three experiments. Cultures of *S. aureus* strains were cultured overnight with inducer ATc (250 ng/ml) and the following day diluted and inoculated into 500 ml of fresh blood with erythromycin and with inducer ATc (250 ng/ml) and incubated at 37°C.
