# Supplementary material for: The AirSR two-component system contributes to Staphylococcus aureus survival in human blood and transcriptionally regulates sspABC operon
Source: Front Microbiol. 2015 Jul 3;6:682. doi: 10.3389/fmicb.2015.00682 (PMC4490255; doi:10.3389/fmicb.2015.00682)
Supplement: Supplementary file 2 [file Presentation_2.PDF]

Figure S2

gi|13700849|dbj|BAB42145.1| (100%), 44,596.5 Da
cysteine protease precursor [Staphylococcus aureus subsp. aureus N315]
39 exclusive unique peptides, 49 exclusive unique spectra, 264 total spectra, 297/393 amino acids (76% coverage)

```
MNSSYKSRVF NIISIIMVSM LILSLGAFAN NNKAKADSHS KQLEINVKSD KVPQKVKDLA
QQQFAGYAKA LDKQSNAKTG KYELGEAFKI YKFNGEEDNS YYYPVIKDGK IVYTLTLSPK
NKDDLNKSKE DMNYSVKISN FIAKDLDQIK DKNSNITVLT DEKGFYFEED GKVRLVKATP
LPGNVKEKES AKTVSAKLKQ ELKNTVTPTK VEENEAIQED QVQYENTLKN FKIREQQFDN
SWCAGFSMAA LLNATKNTDT YNAHDIMRTL YPEVSEQDLP NCSTFPNQMI EYGKSQGRDI
HYQEGVPSYE QVDQLTKDNV GIMILAQSVS QNPNDPHLGH ALAVVGNAKI NDQEKLIYWN
PWDTELSIQD ADSSLLHLSF NRDYNWYGSM IGY
```

Figure S2. Peptides of staphopain B identified by mass spectrometry. Scaffold Viewer version 4.3 was used to analyze and visualize peptide sequences for each sample. Yellow highlighting indicates peptide detection by mass spectrometry. The first 48 amino of the zymogen are cleaved by SspA to activate SspB and were not detected by mass spectrometry.
